# Reel Syndrome: An Atypical Cause for Inappropriate Shocks in a Patient with Automated Implantable Cardioverter Defibrillator (AICD)

**DOI:** 10.7759/cureus.2237

**Published:** 2018-02-27

**Authors:** Rabia Mohammad, Adil Pervaiz, Muhammad Mufti, Khurram Khan, Sarah Syed, Sudhakar Prabhu

**Affiliations:** 1 Internal Medicine, Coney Island Hospital, Brooklyn, USA; 2 Internal Medicine, St. Mary's Medical Center, Long Beach, USA; 3 Department of Cardiology, Coney Island Hospital, Brooklyn, USA

**Keywords:** reel syndrome, twiddler syndrome, ratchet syndrome, aicd, automated implantable cardioverter defibrillator

## Abstract

A 71-year-old woman, with the past medical history of heart failure with reduced ejection fraction (EF) and automated implantable cardioverter defibrillator (AICD) placement (for low EF 5-10%) in 2015, presented in February 2017 with the complaint of AICD shocks following an episode of vomiting. She denied any chest pain, abdominal pain, shortness of breath, palpitation, or dizziness. Electrocardiogram (EKG) on admission showed ectopic atrial rhythm with premature ventricular contractions in bigeminies, anterior fascicular block, and left axis deviation. On examination of the cardiovascular system, there was a normal S1 heart sound with a loud A2. There was no jugular venous distention on the neck or pitting edema on the legs. Laboratory studies showed no elevation of cardiac enzymes. Evaluation with chest x-ray showed the right ventricular lead had migrated to the right atrium and the defibrillator generator was flipped with leads coiled around it in transverse axis. AICD interrogation was performed which revealed inappropriate shocks were due to atrial fibrillation with rapid ventricular rate and loss of capture of the right ventricular lead. The diagnosis of Reel syndrome was made, and an electrophysiologist was consulted for replacement of the AICD.

Reel syndrome is a variant of Twiddler’s syndrome, which is a rare complication of pacemaker implantation. It manifests with the rotation of generator on transverse axis with leads coiling around it. Twiddler’s syndrome, on the other hand, is the rotation of the generator on its long axis, which causes damage to the leads by twisting. Reel syndrome is usually observed within months from the placement of the generator compared to Twiddler, which takes years to occur. Twiddler and Reel's syndromes have similar contributing factors, such as female gender, obesity, large pocket, old age, dementia, and deep brain stimulation. Our patient only had two risk factors, namely, the sex and age.

We propose that every patient with a pacemaker malfunction and AICD shocks should have a posterior-anterior (PA) chest x-ray and a lateral chest x-ray in addition to AICD interrogation. Twiddler’s syndrome is effortlessly observed because of the twisting of dual leads compared to Reel syndrome, which is not straightforward because of the lack tortuosity of the leads.

## Introduction

The presenting patient is a 71-year-old woman with the past medical history of heart failure with reduced ejection fraction and automated implantable cardioverter defibrillator (AICD) placement in 2015. She presented to the emergency room in 2017 after she was shocked twice with the AICD and was noted to have a condition called Reel syndrome.

## Case presentation

A 71-year-old woman with the past medical history of heart failure with reduced ejection fraction (EF) and AICD placement in 2015 (for low EF 5-10%) presented to the emergency room in February 2017 complaining that she had received two shocks from the AICD following an episode of vomiting. Her medical history was significant for hypertension, chronic kidney disease, coronary artery disease, and heart failure with reduced ejection fraction. Surgical history included aortic valve replacement and annular ring placement in the mitral and tricuspid valves.

**Figure 1:**
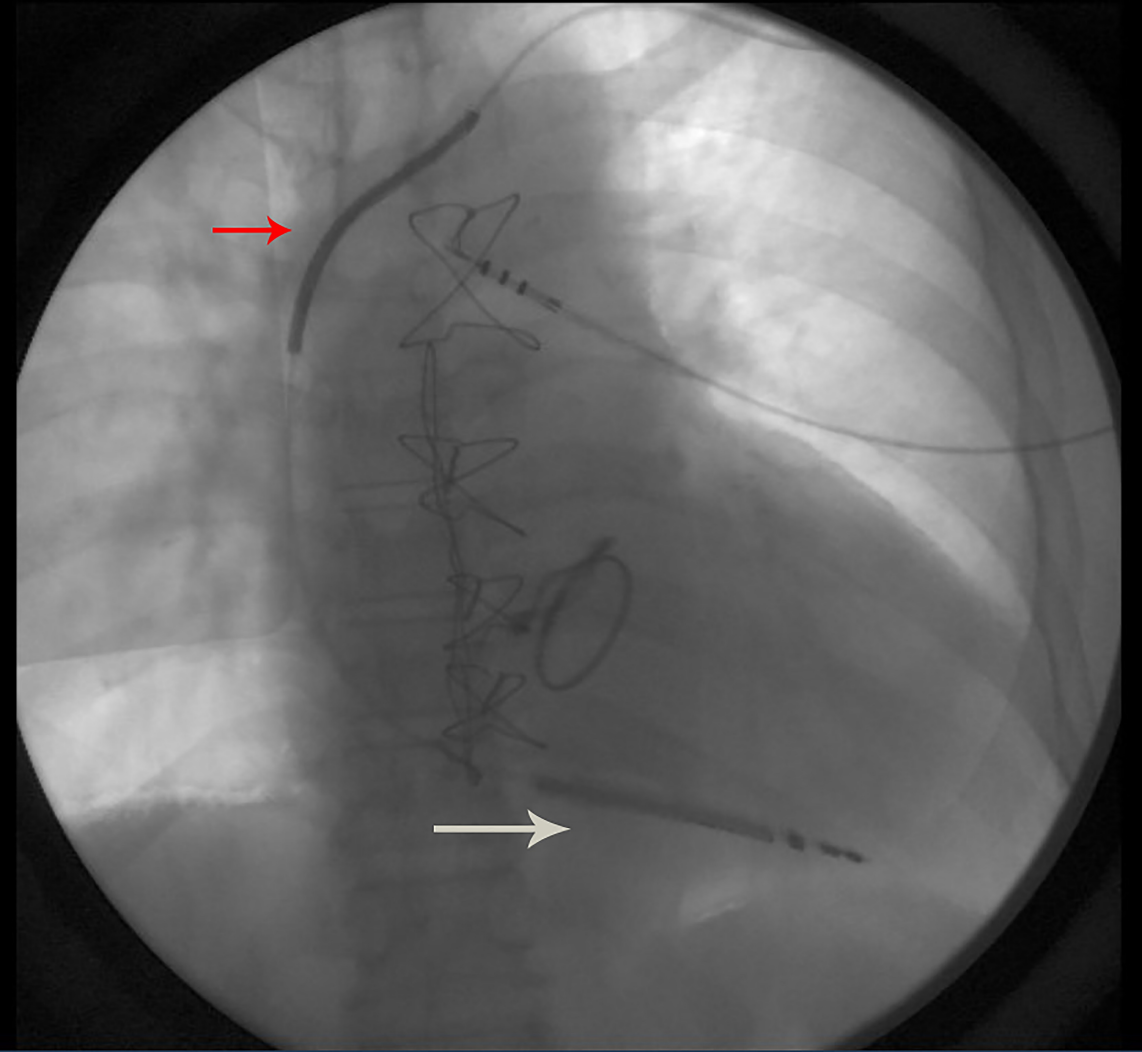
Fluoroscopic confirmation of lead placement Confirmation of lead placement in 2015 with position of right ventricle lead (white arrow) and position of coil (red arrow)

**Figure 2:**
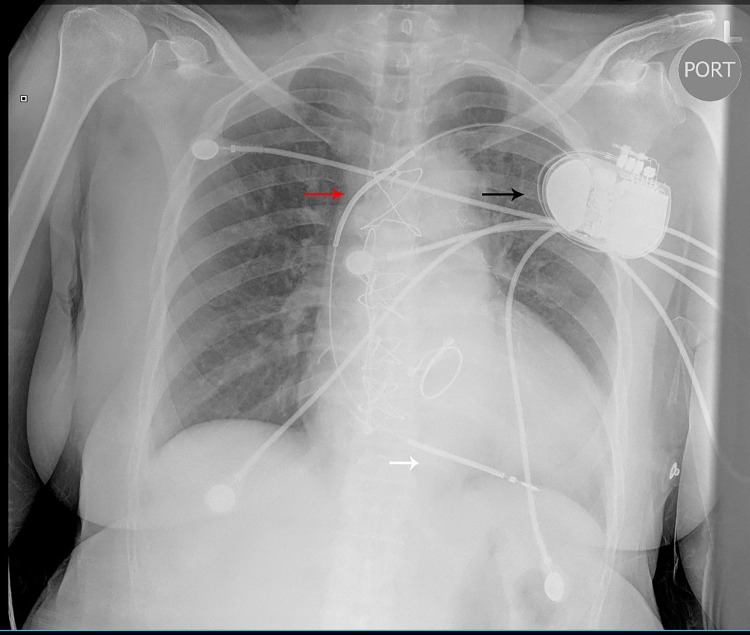
Chest x-ray performed after AICD placement in 2015 Right ventricle lead (white arrow) in the correct place, coil (red arrow) and generator (black arrow). Note its orientation. AICD: automated implantable cardioverter defibrillator

**Figure 3:**
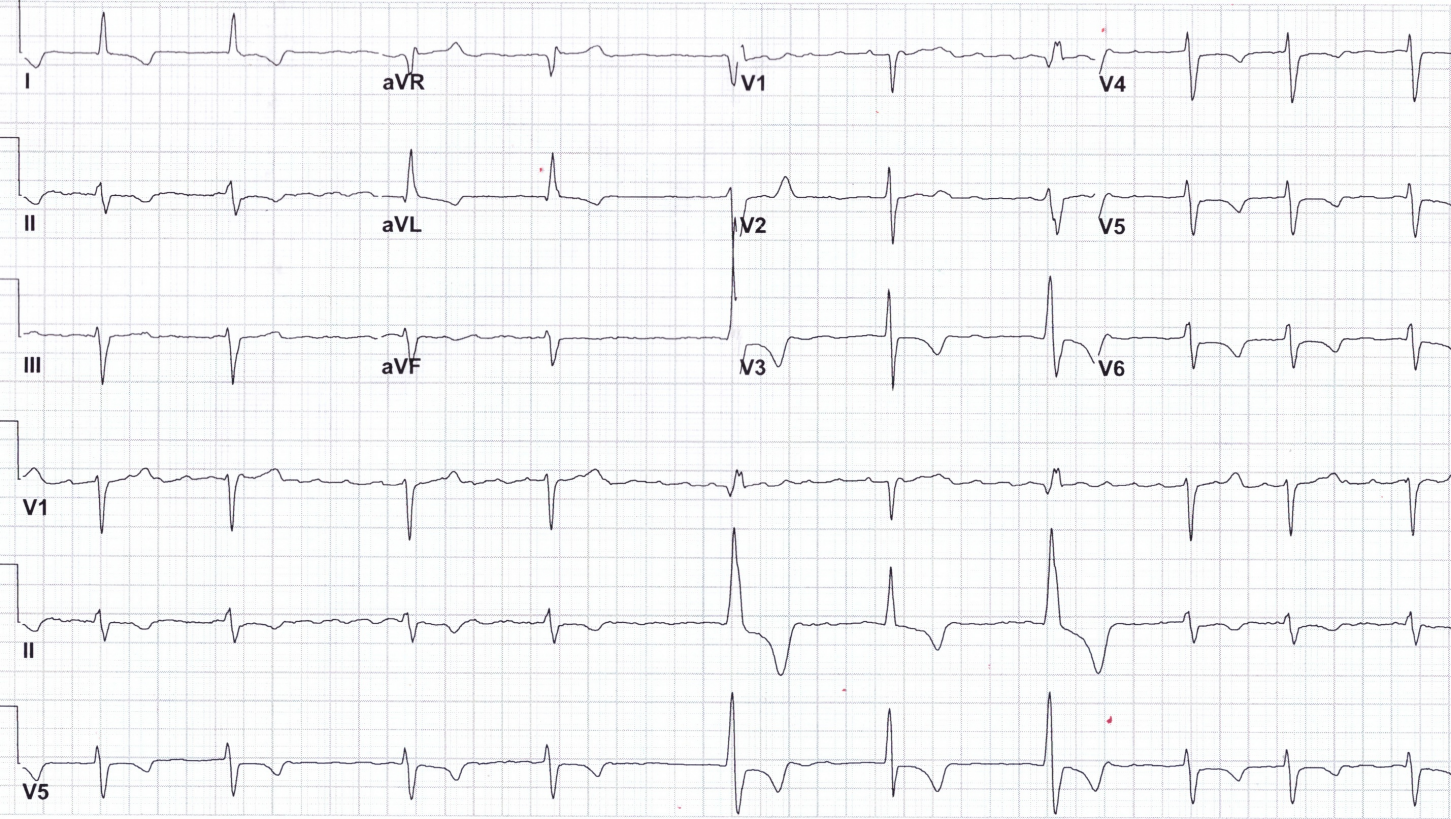
Electrocardiogram on admission

The patient had the AICD placed in August 2015 for the primary prevention of severe systolic dysfunction (Figures 1-2). Since then, she had two previous admissions for AICD shocks in December 2015 and March 2016, which were assessed as inappropriate shocks due to atrial fibrillation with a rapid ventricular response and an otherwise normally functioning AICD on interrogation. Chest x-ray done at that time showed correct placement of leads. In this encounter, she denied any chest pain, abdominal pain, shortness of breath, palpitations, or dizziness. Electrocardiogram (EKG) on admission showed an ectopic atrial rhythm with premature ventricular contractions in bigeminies, an anterior fascicular block, and a left axis deviation (Figure 3). Her labs were significant for normal cardiac enzymes and normocytic normochromic anemia with a hemoglobin of 9.2 g/dL, which was around her baseline. Creatinine was 2.00 mg/dL, and serum potassium was 5.2 mEq/L, which was attributed to chronic kidney disease. Brain natriuretic peptide (BNP) on admission was 2,292 pg/mL, which was lower than her baseline BNP for a past hospital admission for acute decompensation of heart failure.

On examination, she was found to be non-ill appearing, in no acute distress, sitting in bed, and speaking full sentences. She was alert, awake, and oriented to self, person, and time. No jugular venous distension was observed. She had normal S1 heart sounds but loud A2. There was no early diastolic murmur or collapsing pulse. The pacemaker was in the left pectoral area with no erythema, fluid collection, or erosion. The abdomen was soft, non-tender, and bowel sounds were normal. Lower extremities had no pitting edema, and pulses were slightly more diminished than normal pulse (2+). Her recorded blood pressure was 125/83 mm-Hg with a pulse rate of 87 beats per minute. Her respiratory rate was about 16 breaths per minute, and her body mass index at this encounter was recorded at 28.

**Figure 4:**
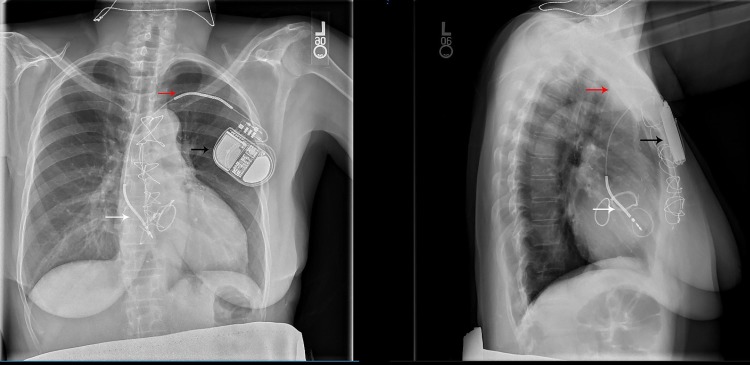
Posterior-anterior chest x-ray taken in 2017 Note the position of the right ventricle lead (white arrow) and coil (red arrow). The generator (black arrow) is flipped.

The patient’s AICD interrogation was assessed as an inappropriate shock due to atrial fibrillation with a rapid ventricular rate. It also revealed loss of capture of the right ventricular lead due to lead migration. Further evaluation with a chest x-ray showed that the right ventricular lead had migrated to the right atrium (Figure 4). The defibrillator generator had flipped with leads coiled around it in the transverse axis. No apparent lead damage was observed. The patient was diagnosed with Reel syndrome, and an electrophysiologist was consulted for replacement of the AICD.

## Discussion

Reel syndrome is a variant of Twiddler’s syndrome, which is a rare complication of pacemaker implantation. Twiddler’s syndrome was first described in 1968 by Dr. Colin E. Bayliss [1]. Initially, the syndrome was identified as lead retraction due to the rotation of the pulse generator. The cause of a generator rotation can be spontaneous or intentional [1]. Twiddler’s syndrome is the rotation of the generator on its long axis, which causes damage to the leads; if it’s a dual lead, the x-ray will show tangling of the leads. In comparison, there is a rotation of the generator on its transverse axis in Reel syndrome but no damage to the leads. The leads will be coiled around the generator and retracted. Reel syndrome is usually observed within months from the placement of the generator compared to Twiddler which takes years to occur [2].

Twiddler and Reel's syndromes have similar contributing factors, such as female gender, obesity, large pocket, old age, dementia, and deep brain stimulation [3-4]. The patient we described was neither obese nor had a diagnosis of dementia. She was a female in the geriatric population, giving her two of the risk factors. In Reel syndrome, the leads aren’t fractured and are working well so the patient will still get shocked; however, in this case, the patient was getting shocked based on an atrial arrhythmia and not ventricular arrhythmia.

Currently, studies are being done to prevent Twiddler and Reel syndrome from occurring via anchoring of the leads. A case series of 349 patients, who underwent deep brain stimulations, was done to see if dual lead anchoring decreased the likelihood of developing Twiddler’s syndrome. There were only three cases out of 349 with deep brain stimulation who developed Twiddler’s syndrome and needed a new generator. In this study, they found no patient with two anchoring holes who developed Twiddler’s syndrome [4].

Twiddler and Reel syndromes should be considered in patients with a history of inappropriate or appropriate shocks. Preventive measures of a smaller generator pocket, compression band around the upper chest and shoulder for seven days, and close follow-up with a chest x-ray should be considered to decrease the incidence of this syndrome [5]. Annual posterior-anterior (PA) and lateral chest x-rays can be performed for patients with multiple risk factors to catch early onset of these syndromes and prevent a patient from getting inappropriate shocks.

## Conclusions

In conclusion, we propose that every patient with pacemaker malfunction and AICD shocks should have a posterior-anterior and lateral chest x-ray, which should be compared to the x-ray taken after generator placement, to rule out any lead migration. Twiddler’s syndrome is effortlessly observed because of the twisting of dual leads. Reel syndrome is not straightforward because of the lack tortuosity of the leads. A smaller generator pocket, compression band around the upper chest and shoulder for seven days, and close follow-up with a chest x-ray should be considered. Annual PA and lateral chest x-rays can be performed for patients with multiple risk factors for Twiddler and Reel syndrome to prevent them from getting inappropriate shocks.
